# Nomograms predict survival benefits of radical prostatectomy and chemotherapy for prostate cancer with bone metastases: A SEER‐based study

**DOI:** 10.3389/fonc.2022.1020898

**Published:** 2022-12-06

**Authors:** Donglin Sun, Ao Lin, Zhun Sun, Shuqi Yang, Yuexin Sun, Anning Chen, Guojun Qian, Zhonghua Ji, Li Wang

**Affiliations:** ^1^ Center for Cancer and Immunology Research, State Key Laboratory of Respiratory Disease, Affiliated Cancer Hospital and Institute of Guangzhou Medical University, Guangzhou, China; ^2^ The State Key Lab of Respiratory Disease, Institute of Public Health, Guangzhou Medical University, Xinzao, Guangzhou, China; ^3^ Department of Clinical Medicine, The Second Clinical School of Guangzhou Medical University, Guangzhou, China; ^4^ Department of Anesthesia, Shanghai East Hospital, Tongji University School of Medicine, Shanghai, China; ^5^ Nephrology Department, Southern Medical University Affiliated Longhua People’s Hospital, Shenzhen, China

**Keywords:** prostate cancer, bone metastasis, prognosis, nomogram, radical prostatectomy, chemotherapy

## Abstract

**Purpose:**

This study aimed to identify independent prognosis-associated factors of bone-metastatic prostate cancer. The nomograms were further developed to obtain indicators for the prognostic evaluation.

**Methods:**

A total of 7315 bone-metastatic prostate cancer (PCa) patients from 2010 to 2016 were retrospectively collected from the Surveillance, Epidemiology, and End Results (SEER) database. Patients were randomly divided into the training cohort (n=5,120) and test cohort (n=2,195) in a ratio of 7:3. Univariate and multivariate Cox regression models were applied to evaluate potential risk factors. A 1:1 propensity score matching (PSM) was further performed to decrease the confounding effect and re-evaluate the influence of radical prostatectomy and chemotherapy on prognosis. Combining these potential prognosis factors, the nomograms of cancer-specific survival (CSS) and overall survival (OS) at different times were established. C-indexes, calibration curves, and decision curves were developed to evaluate the discrimination, calibration, and clinical benefit of the nomograms.

**Results:**

Eleven independent prognosis factors for CSS and twelve for OS were utilized to conduct the nomograms respectively. The C-indexes of nomograms for CSS and OS were 0.712 and 0.702, respectively. A favorable consistency between the predicted and actual survival probabilities was demonstrated by adopting calibration curves. Decision curves also exhibited a positive clinical benefit of the nomograms.

**Conclusions:**

Nomograms were formulated successfully to predict 3-year and 5-year CSS and OS for bone-metastatic PCa patients. Radical prostatectomy and chemotherapy were strongly associated with the bone-metastatic PCa prognosis.

## Introduction

Prostate cancer (PCa) is an epithelial malignant tumor that occurs in the prostate. It remains the most common lethal malignancy diagnosed among men in the United States and the second leading cause of male cancer mortality (1). There were 248,530 newly diagnosed cases in 2021 in the United States, and 34,130 men died of prostate cancer.

Due to the deficiency of typical clinical symptoms in the early stages, prostate cancer is usually detected in the middle and late stages when it spreads to multiple organs in the body (2). Bone is the most frequent site of metastases (3). Approximately 10% of new PCa patients are diagnosed with bone metastasis, increasing to 80% at advanced stages (4). Suffering from bone pain, spinal cord compression, and pathological fractures, most PCa patients diagnosed with bone metastasis undergo severe economic burdens and increasing mortality risks (5).

The systematic treatments for bone-metastatic PCa patients need to be individualized based on patient-specific factors, including endocrine therapy, chemotherapy, radiotherapy, and surgery for primary or metastatic sites (6). Despite the advancement in understanding the bone-metastatic mechanism, it is still clinically controversial as to which therapy should be applied among patients for a better survival rate (7, 8). Therefore, our aim of this study is to excavate characteristics and possible prognostic factors of patients with bone-metastatic PCa as comprehensively as possible, and specially to analyze prognosis differences among different clinical treatments from the perspective of overall survival and cancer-specific survival using Surveillance, Epidemiology, and End Results (SEER) data.

## Material and source

### Data source

All patients’ information was obtained from the Surveillance, Epidemiology, and End Results (SEER) database, which covers approximately 26.5% of the U.S. population. This data is based on cancer incidence in 17 registries across the United States between 2000 and 2019, submitted in Nov 2021, and released in April 2022. SEER*Stat 8.4.0 was used to filter and collect subjects’ information.

### Cohort selection

**Figure 1:**
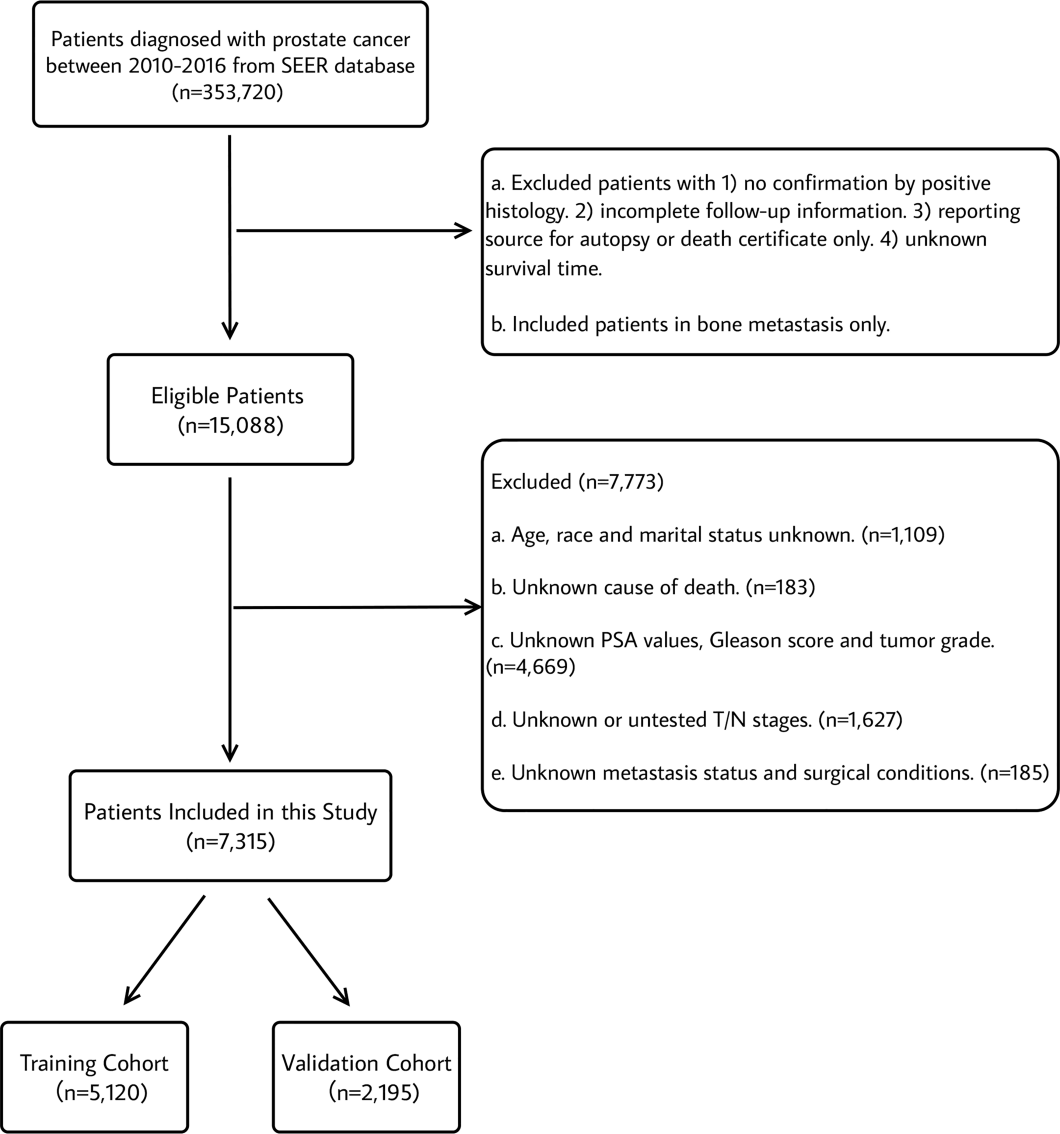
Flowchart describing the selection of patients in the Surveillance, Epidemiology, and End Results (SEER) database, 2010–2016.

This study population included all patients diagnosed with bone metastasis in prostate cancer (International Classification of Disease for Oncology (ICD-O-3) code C61.9) from 2010-2016 with at least 3-year active follow-up data. Pathologic tumor stage was recorded according to the American Joint Committee on Cancer (AJCC) 7th TNM staging system of prostate cancer. The inclusion and exclusion criteria were displayed in **Figure 1**. The inclusion criteria were as follows: (1) Diagnosis of prostate cancer (primary sites: C61.9 and ICD-O-3); (2) diagnosed between 2010 and 2016 with at least 3-year follow-up data; (3) known survival months and specific causes of death; (4) diagnostic confirmation based on positive histology; (5) diagnosed in bone metastasis; (6) known age, marital status and ethnicity; (7) known 7th American Joint Committee on Cancer (AJCC) TNM stage, 7th AJCC T status and 7th AJCC N status at diagnosis; (8) known PSA values, Gleason scores and tumor grade at diagnosis; and (10) known metastasis status and surgical conditions. A total of 7,315 patients fulfilled the criteria and were randomized with a 7:3 ratio into a training cohort (n=5,120) and a test cohort (n=2,195).

### Study Variables

Study variables extracted from the SEER database include age at diagnosis, race, marital status, tumor differentiation grade, Gleason score, PSA values, 7th American Joint Committee stage (T/N stage), surgical conditions, radiotherapy, chemotherapy, and metastasis status.

**Figure 2:**
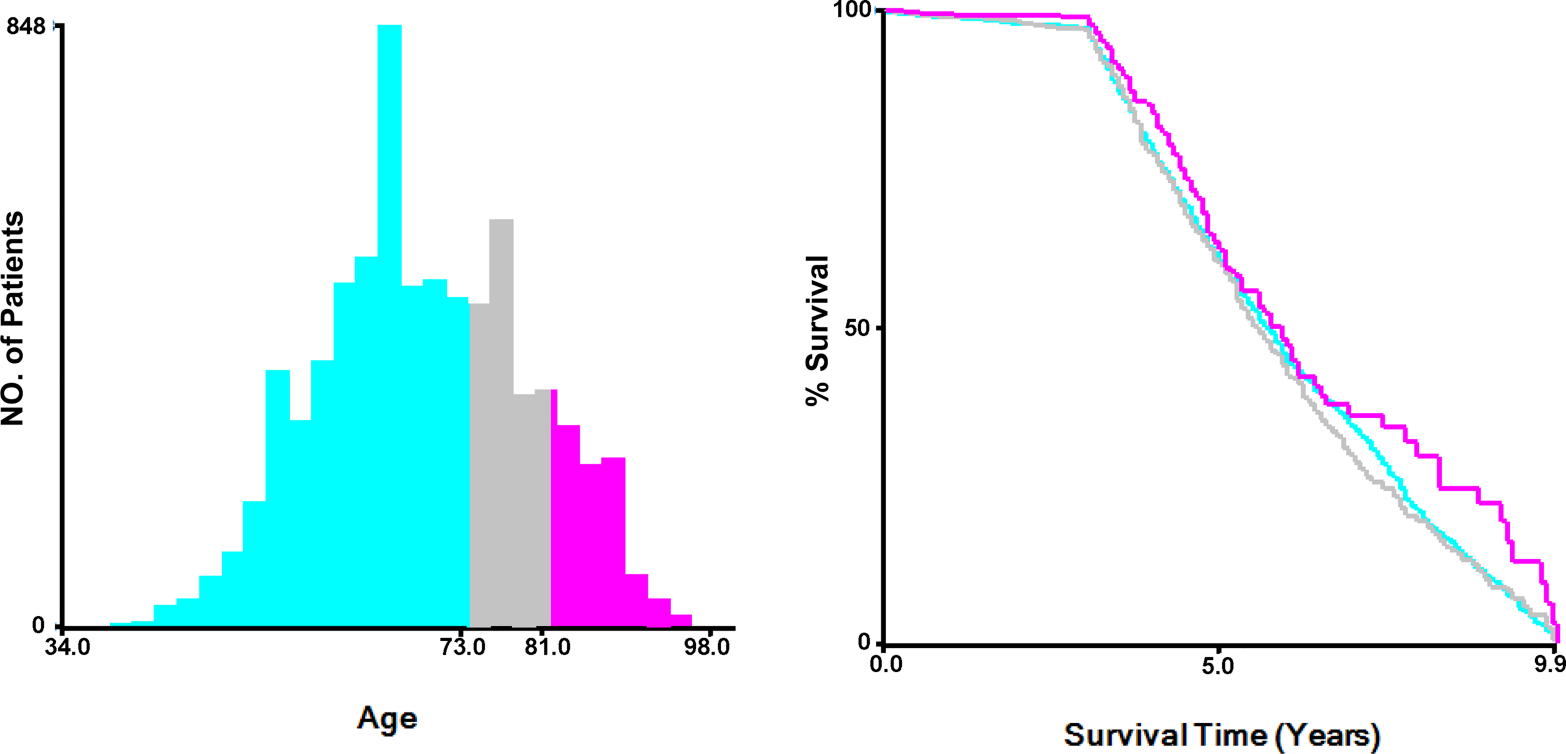
Determination of the optimal cut-off values of age at diagnosis by X-tile.

The optimal cut-off values of the age group were calculated by X-tile software (Yale University, USA), and they were ≤73 years old, 74-81 years old, or >81 years old (**Figure 2**). Categorical variables including race (White, black, and others), marital status (Married, single, and others), tumor grade (Low - grade I well differentiated and grade II moderately differentiated, high - grade III poorly differentiated and grade IV undifferentiated), Gleason Score (≤7, 8, 9, and 10), PSA values (<20ng/ml, 25-50ng/ml, and >50ng/ml), T stage (T1/T2 and T3/T4), N stage (N0 negative and N1 positive), surgery (No surgery of primary site, focal therapy, and radical prostatectomy), radiotherapy (Yes, no/unknown), chemotherapy (Yes, no/unknown), liver metastasis (Yes, no), brain metastasis (Yes, no) and lung metastasis (Yes, no) were reported in this study.

### Endpoint definition

Cancer specific survival (CSS) and overall survival (OS) were defined as primary endpoints. CSS was defined from the date of diagnosis to the date of death caused by prostate cancer, and OS was measured from the date of diagnosis to the date of death occurring as a result of all causes.

### Statistical analysis

All eligible cases were randomized in a 7:3 ratio into training cohort and test cohort. Descriptive statistics were used to compare the baseline characteristics between the training cohort and test cohort through a Chi-squared test. Univariate and multivariate Cox regression models were performed in the training cohort to determine the independent prognostic factors associated with OS and CSS in PCa patients with bone metastasis (9). Survival curves were plotted by the Kaplan-Meier method and compared by the log-rank test. A 1:1 propensity score matching (PSM) in the x method with a 0.02 calipers value was applied to reduce the confounding effect caused by other factors and reevaluate the survival benefits of different treatment patterns (10).

The nomograms for three years and five years of OS and CSS were finally built by combining the prognosis-association factors (11). Internal and external validations were performed by comparing the Harrell concordance indexes (C-index), calibration curves, and decision curves of training and test cohorts. Specifically, the calibration plots with 1,000 resample of bootstrapping were generated by comparing the predicted probability with the actual survival probability; the decision curve analysis (DCA) was used for identifying the clinical usefulness of the nomograms.

All statistical analyses were performed by R version (version 4.0.2, R Foundation for Statistical Computing, Vienna, Austria). Two-side *P*-value < 0.05 was considered statistically significant.

## Results

### Patients characteristics

A total of 7,315 eligible male patients with prostate cancer in bone metastasis were collected from the SEER database from 2010-2016. Among them, 5,120 patients were in the training cohort, and 2,195 were in the test cohort. The sociodemographic and clinicopathologic characteristics of all patients were summarized in **Table 1**. There were no statistically significant differences between the training cohort and test cohort except for the tumor grade. 82.93% (4282/5163) of total patients died of prostate cancer by the end of follow up. The median of CSS and OS were 42 months and 35 months, respectively.

**Table 1:** Baseline characteristics of the 7,135 patients with bone metastatic prostate cancer between 2010 and 2016 from the SEER database.

Variables	Total Patients	Training Cohort	Test Cohort	*P*
		(n=7,315)	(n=5,120)		(n=2,195)	
**Age, n(%)**				0.062
≤ 73yrs	4744 (64.9)	3290 (64.3)	1454 (66.2)	
74-81yrs	1525 (20.8)	1066 (20.8)	459 (20.9)	
> 81yrs	1046 (14.3)	764 (14.9)	282 (12.8)	
**Race, n(%)**				0.875
White	5619 (76.8)	3925 (76.7)	1694 (77.2)	
Black	1230 (16.8)	865 (16.9)	365 (16.6)	
Others	466 (6.4)	330 (6.4)	136 (6.2)	
**Marital Status, n(%)**				0.162
Married	4595 (62.8)	3194 (62.4)	1401 (63.8)	
Single	1233 (16.9)	891 (17.4)	342 (15.6)	
Others	1487 (20.3)	1035 (20.2)	452 (20.6)	
**Grade, n(%)**				0.431
Low	729 (10.0)	520 (10.2)	209 (9.5)	
High	6586 (90.0)	4600 (89.8)	1986 (90.5)	
**Gleason Score, n(%)**				0.414
≤ 7	1258 (17.2)	870 (17.0)	388 (17.7)	
8	1802 (24.6)	1244 (24.3)	558 (25.4)	
9	3459 (47.3)	2454 (47.9)	1005 (45.8)	
10	796 (10.9)	552 (10.8)	244 (11.1)	
**PSA (ng/ml), n(%)**				0.965
< 20	1798 (24.6)	1259 (24.6)	539 (24.6)	
20 - 50	1343 (18.4)	936 (18.3)	407 (18.5)	
> 50	4174 (57.1)	2925 (57.1)	1249 (56.9)	
**Surgery, n(%)**				0.608
No	6288 (86.0)	4409 (86.1)	1879 (85.6)	
Focal Therapy	833 (11.4)	572 (11.2)	261 (11.9)	
Radical Prostatectomy	194 (2.7)	139 (2.7)	55 (2.5)	
**Radiation, n(%)**				0.756
No/Unknown	5581 (76.3)	3912 (76.4)	1669 (76.0)	
Yes	1734 (23.7)	1208 (23.6)	526 (24.0)	
**Chemotherapy, n(%)**				1.000
No/Unknown	6140 (83.9)	4298 (83.9)	1842 (83.9)	
Yes	1175 (16.1)	822 (16.1)	353 (16.1)	
**Brain Metastasis, n(%)**				0.749
No	7277 (99.5)	5092 (99.5)	2185 (99.5)	
Yes	38 (0.5)	28 (0.5)	10 (0.5)	
**Liver Metastasis, n(%)**				0.830
No	7102 (97.1)	4969 (97.1)	2133 (97.2)	
Yes	213 (2.9)	151 (2.9)	62 (2.8)	
**Lung Metastasis, n(%)**				0.774
No	6912 (94.5)	4841 (94.6)	2071 (94.4)	
Yes	403 (5.5)	279 (5.4)	124 (5.6)	
**T Stage, n(%)**				0.010
T1/T2	6412 (87.7)	4454 (87.0)	1958 (89.2)	
T3/T4	903 (12.3)	666 (13.0)	237 (10.8)	
**N Stage, n(%)**				0.603
N0	5032 (68.8)	3532 (69.0)	1500 (68.3)	
N1	2283 (31.2)	1588 (31.0)	695 (31.7)	

### Identification of independent prognostic factors

Univariate and multivariate Cox regression analysis were performed to analyze independent prognostic factors related with CSS and OS of PCa patients with bone metastases. We found age, race, marital status, Gleason score, PSA value, tumor grade, surgery, chemotherapy, live metastasis, lung metastasis, and T stage were associated with both CSS and OS (**Table 2**). Specifically, compared with patients without any surgery taken, patients with radical prostatectomy indicated a significantly superior CSS and OS (P < 0.001). While patients who underwent focal therapy, including ablation, cryotherapy, and tissue destruction, appeared to have higher cancer-specific and overall mortality risks (P < 0.001). Bone-metastatic PCa patients with chemotherapy treatment had a better CSS and OS (P < 0.001 and P = 0.001, respectively) than those without chemotherapy therapy or in unknown chemotherapy status. In contrast, no significant differences in radiation treatment were observed in either CSS or OS.

**Table 2:** Univariate and multivariate analysis of CSS and OS for PCa patients with BM.

Variables	Prostate Cancer-Specific Survival				Overall Survival			
	Univariate Analysis		Multivariate analysis		Univariate Analysis		Multivariate analysis	
	HR (95% CI)	*P*	HR (95% CI)	*P*	HR (95% CI)	*P*	HR (95% CI)	*P*
**Age**								
≤ 73yrs	Reference		Reference		Reference		Reference	
74-81yrs	**1.3 (1.19-1.42)**	**< 0.001**	**1.29 (1.18 - 1.41)**	**< 0.001**	**1.36 (1.26 - 1.48)**	**< 0.001**	**1.33 (1.23 - 1.45)**	**< 0.001**
> 81yrs	**1.62 (1.46-1.79)**	**< 0.001**	**1.52 (1.37 - 1.69)**	**< 0.001**	**2.02 (1.85 - 2.20)**	**< 0.001**	**1.87 (1.71 - 2.05)**	**< 0.001**
**Race**								
White	Reference		Reference		Reference		Reference	
Black	1.02 (0.93 - 1.12)	0.66	0.99 (0.90 - 1.10)	0.912	1.01 (0.92 - 1.10)	0.854	1.01 (0.92 - 1.10)	0.907
Others	**0.72 (0.61 - 0.84)**	**< 0.001**	**0.69 (0.59 - 0.81)**	**< 0.001**	**0.71 (0.61 - 0.82)**	**< 0.001**	**0.68 (0.59 - 0.79)**	**< 0.001**
**Marital Status**								
Married	Reference		Reference		Reference		Reference	
Single	**1.20 (1.09 - 1.32)**	**< 0.001**	**1.14 (1.03 - 1.26)**	**0.009**	**1.20 (1.09 - 1.31)**	**< 0.001**	**1.17 (1.07 - 1.28)**	**< 0.001**
Others	**1.19 (1.09 - 1.31)**	**< 0.001**	1.04 (0.95 - 1.14)	0.396	**1.26 (1.16 - 1.37)**	**< 0.001**	1.09 (1.00 - 1.18)	0.052
**Grade**								
Low	Reference		Reference		Reference		Reference	
High	**2.06 (1.78-2.40)**	**< 0.001**	**1.46 (1.20 - 1.79)**	**< 0.001**	**1.91 (1.68-2.18)**	**< 0.001**	**1.48 (1.24 - 1.76)**	**< 0.001**
**Gleason Score**								
≤ 7	Reference		Reference		Reference		Reference	
8	**1.22 (1.08 - 1.39)**	**0.003**	0.89 (0.76 - 1.05)	0.171	**1.17 (1.04 - 1.31)**	**0.008**	**0.86 (0.74 - 0.99)**	**0.030**
9	**2.01 (1.79 - 2.25)**	**< 0.001**	**1.44 (1.24 - 1.67)**	**< 0.001**	**1.81 (1.64 - 2.00)**	**< 0.001**	**1.29 (1.13 - 1.47)**	**< 0.001**
10	**3.13 (2.72 - 3.60)**	**< 0.001**	**2.20 (1.85 - 2.62)**	**< 0.001**	**2.68 (2.36 - 3.04)**	**< 0.001**	**1.88 (1.61 - 2.19)**	**< 0.001**
**PSA (ng/ml)**								
< 20	Reference		Reference		Reference		Reference	
20 - 50	**1.21 (1.08 - 1.36)**	**0.001**	1.07 (0.95 - 1.21)	0.272	**1.25 (1.13 - 1.40)**	**< 0.001**	1.10 (0.99 - 1.23)	0.073
> 50	**1.61 (1.47 - 1.76)**	**< 0.001**	**1.32 (1.20 - 1.45)**	**< 0.001**	**1.62 (1.49 - 1.76)**	**< 0.001**	**1.35 (1.24 - 1.47)**	**< 0.001**
**Surgery**								
No	Reference		Reference		Reference		Reference	
Focal Therapy	**1.48 (1.33 - 1.64)**	**< 0.001**	**1.24 (1.12 - 1.39)**	**< 0.001**	**1.50 (1.37 - 1.66)**	**< 0.001**	**1.25 (1.14 - 1.38)**	**< 0.001**
Radical Prostatectomy	**0.20 (0.14 - 0.30)**	**< 0.001**	**0.30 (0.20 - 0.45)**	**< 0.001**	**0.23 (0.16 - 0.32)**	**< 0.001**	**0.34 (0.24 - 0.48)**	**< 0.001**
**Radiation**								
No/Unknown	Reference		Reference		Reference		Reference	
Yes	0.98 (0.90 - 1.07)	0.663	1.01 (0.92 - 1.10)	0.871	0.96 (0.88 - 1.03)	0.252	1.00 (0.93 - 1.08)	0.958
**Chemotherapy**								
No/Unknown	Reference		Reference		Reference		Reference	
Yes	0.96 (0.87 - 1.07)	0.458	**0.89 (0.80 - 0.99)**	**0.031**	0.86 (0.79 - 0.95)	**0.003**	**0.85 (0.77 - 0.94)**	**0.001**
**Brain Metastasis**								
No	Reference		Reference		Reference		Reference	
Yes	**2.50 (1.64 - 3.80)**	**< 0.001**	1.52 (0.98 - 2.37)	0.062	**2.35 (1.58 - 3.48)**	**< 0.001**	**1.61 (1.07 - 2.43)**	**0.024**
**Liver Metastasis**								
No	Reference		Reference		Reference		Reference	
Yes	**3.08 (2.58 - 3.69)**	**< 0.001**	**2.59 (2.14 - 3.14)**	**< 0.001**	**2.74 (2.31 - 3.25)**	**< 0.001**	**2.42 (2.02 - 2.91)**	**< 0.001**
**Lung Metastasis**								
No	Reference		Reference		Reference		Reference	
Yes	**1.68 (1.46 - 1.94)**	**< 0.001**	**1.51 (1.30 - 1.77)**	**< 0.001**	**1.53 (1.33 - 1.75)**	**< 0.001**	**1.42 (1.23 - 1.64)**	**< 0.001**
**T Stage**								
T1/T2	Reference		Reference		Reference		Reference	
T3/T4	**1.52 (1.37-1.68)**	**< 0.001**	**1.22 (1.10 - 1.35)**	**< 0.001**	**1.46 (1.33-1.60)**	**< 0.001**	**1.20 (1.09 - 1.10)**	**< 0.001**
**N Stage**								
N0	Reference		Reference		Reference		Reference	
N1	**1.22 (1.13 - 1.32)**	**< 0.001**	1.06 (0.98 - 1.15)	0.150	**1.13 (1.05 - 1.21)**	**0.001**	1.02 (0.94 - 1.10)	0.653

Bold values significant of P values (P< 0.05).

**Figure 3:**
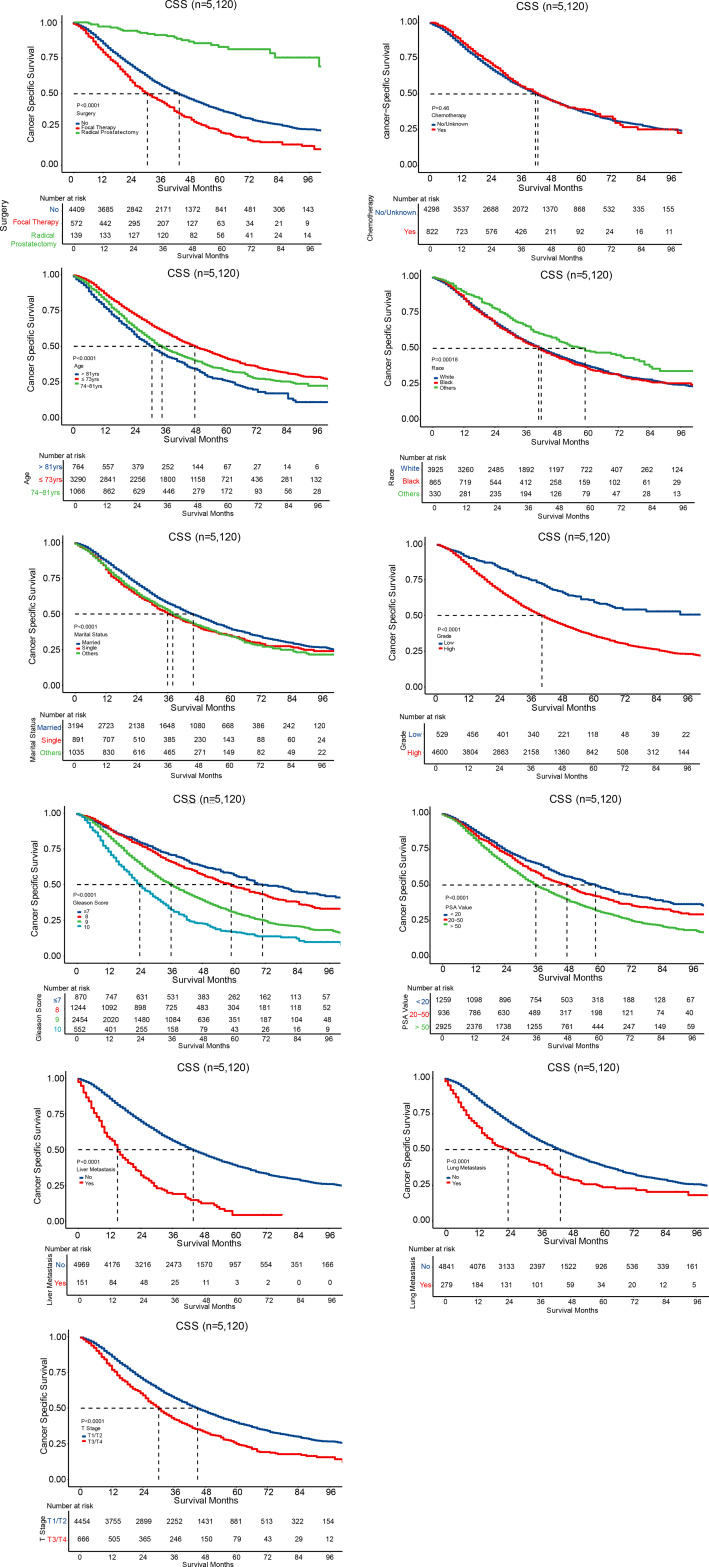
Survival curves for CSS.

**Figure 4:**
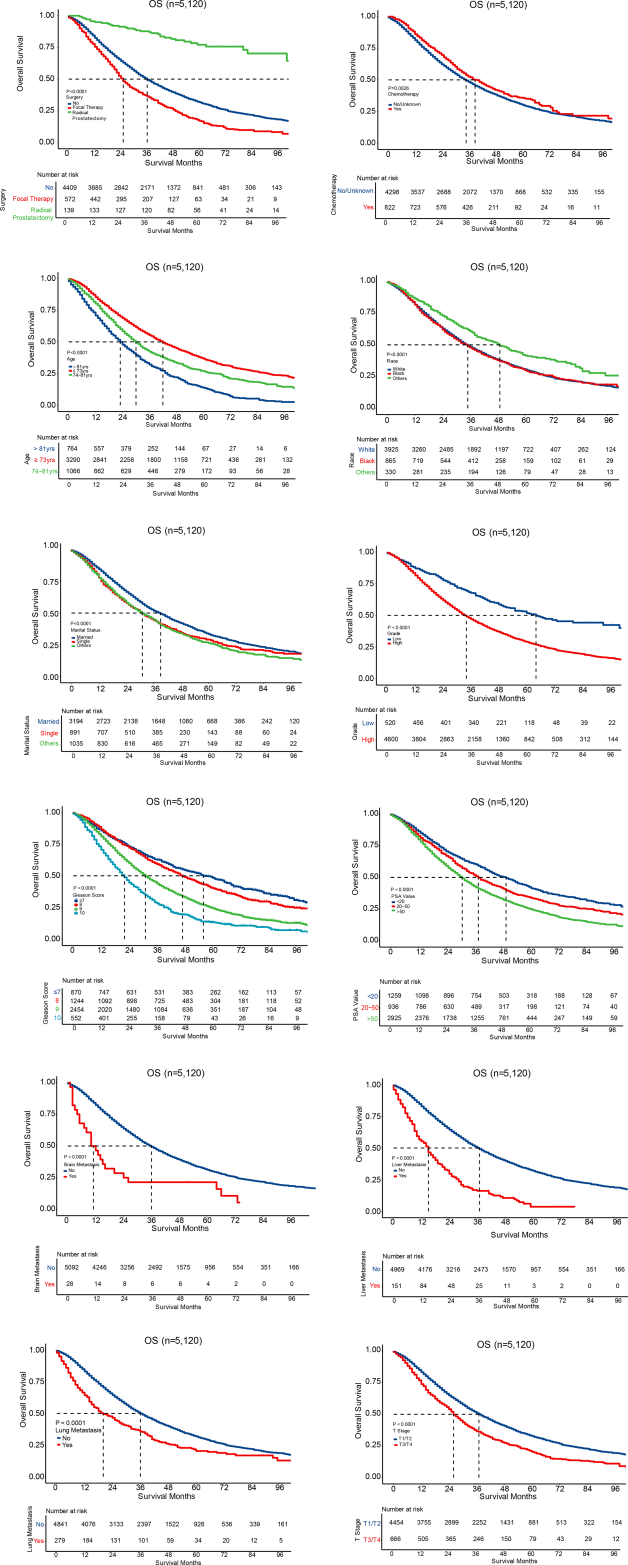
Survival curves for OS.

All the independent prognostic factors on CSS (**Figure 3**) and OS (**Figure 4**) were demonstrated by Kaplan–Meier curves, including age, race, marital status, grade, Gleason score, PSA value, liver metastasis, brain metastasis, lung metastasis, T stage, surgery, and chemotherapy. Patients with radical prostatectomy were shown to comparatively increase both CSS and OS (both P < 0.001), reaching 3-year CSS and OS of 91.1% and 87.7%, respectively as well as 5-year CSS and OS of 82.8% and 77.1%, respectively. Chemotherapy was only associated to impact overall survival (P = 0.0026), while no survival advantage was revealed on CSS (P=0.46) in Kaplan-Meier curves.

### Identification of prognostic factors of OS and CSS in 1:1 matched group by PSM

To further evaluate the impact of treatment patterns on OS and CSS in PCa patients with bone metastasis, we executed chemotherapy stratification on all patient parameters (**Supplementary Tables 1**, **2**). Also, a 1:1 ratio paired cohort matching was performed by propensity score matching (PSM) to minimize the selection bias for variables including age, race, marital status, surgery, tumor grade, Gleason score, PSA values, metastasis conditions, T/N stage. After PSM, there were no remarkable differences in each confounding factor on either chemotherapy or radical prostatectomy stratification (**Supplementary Tables 3**, **4**). Through the univariate and multivariate Cox regression analysis after PSM, we found radical prostatectomy and chemotherapy were still significantly associated with survival benefits on both CSS and OS among bone-metastatic PCa patients (**Supplementary Tables 5**, **6**).

### Development and validation of a prognostic nomogram for CSS and OS

**Figure 5:**
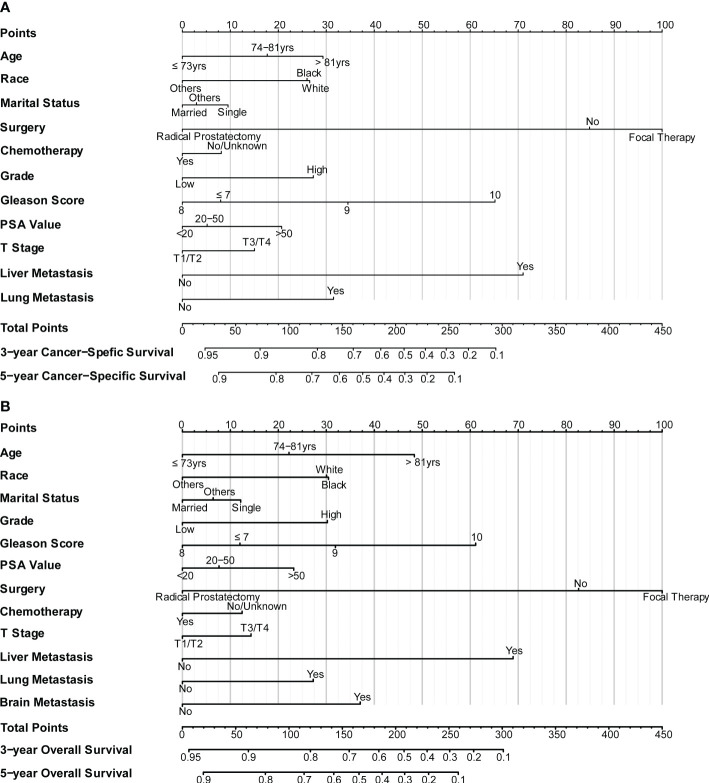
**(A)** Nomogram for CSS in patients of bone-metastatic prostate cancer; **(B)** Nomogram for OS in patients of bone-metastatic prostate cancer.

**Figure 6:**
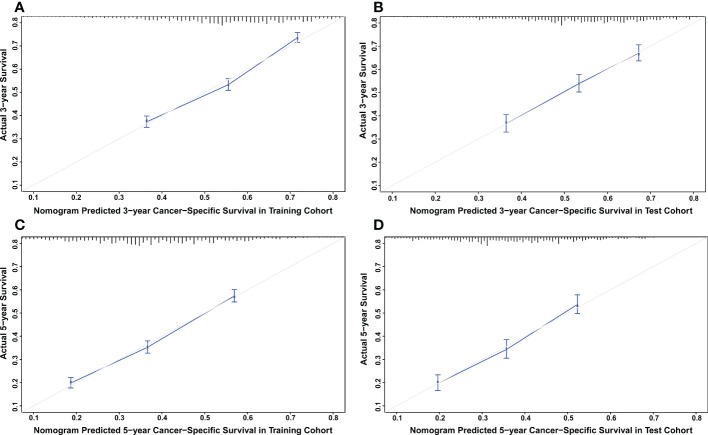
**(A)** Calibration plot for CSS at 3 years in the training cohort. **(B)** Calibration plot for CSS at 3 years in the test cohort. **(C)** Calibration plot for CSS at 5 years in the training cohort. **(D)** Calibration plot for CSS at 5 years in the test cohort.

**Figure 7:**
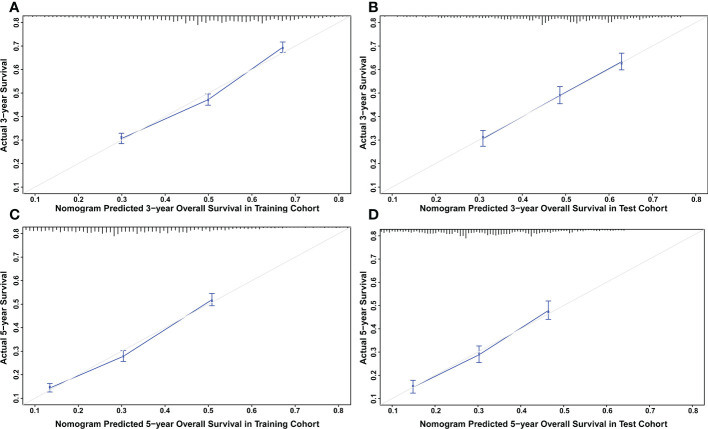
**(A)** Calibration plot for OS at 3 years in the training cohort. **(B)** Calibration plot for OS at 3 years in the test cohort. **(C)** Calibration plot for OS at 5 years in the training cohort. **(D)** Calibration plot for OS at 5 years in the test cohort.

**Figure 8:**
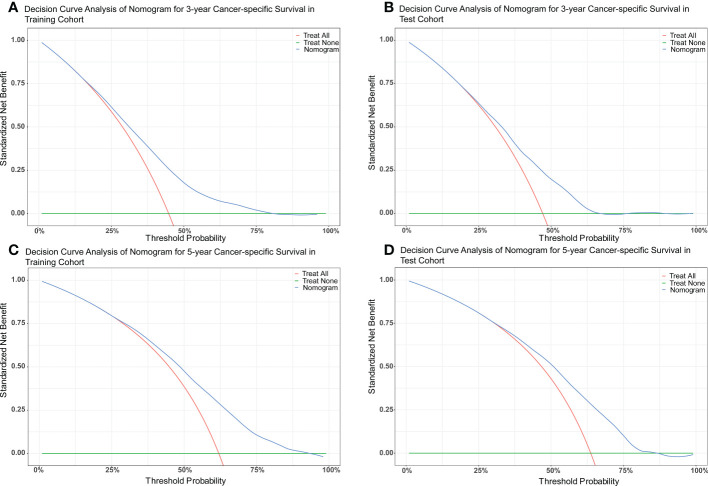
**(A)** DCA of nomogram for 3-year CSS in the training cohort. **(B)** DCA of nomogram for 3-year CSS in the test cohort. **(C)** DCA of nomogram for 5-year CSS in the training cohort. **(D)** DCA of nomogram for 5-year CSS in the test cohort.

**Figure 9:**
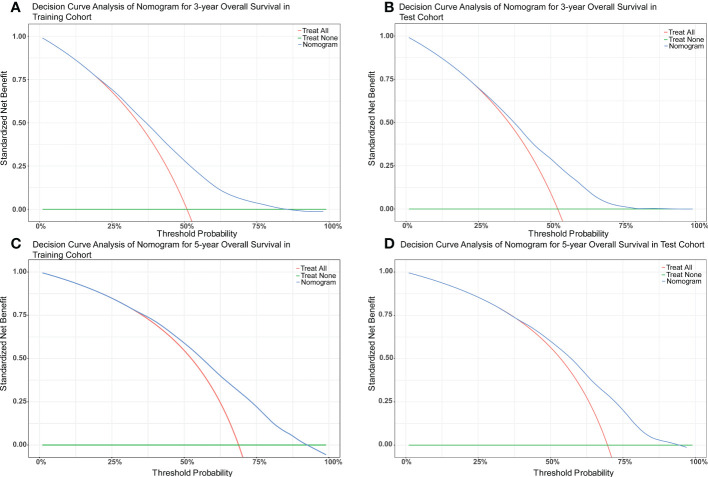
**(A)** DCA of nomogram for 3-year OS in the training cohort. **(B)** DCA of nomogram for 3-year OS in the test cohort. **(C)** DCA of nomogram for 5-year OS in the training cohort. **(D)** DCA of nomogram for 5-year OS in the test cohort.

The nomograms were constructed to predict 3-year and 5-year CSS and OS in the training cohort of PCa patients with bone metastasis based on independent prognostic factors from multivariate Cox analysis (**Figure 5**). The C-index for nomogram of CSS and OS was 0.712 (95% CI, 0.701-0.723) and 0.702 (95% CI, 0.691 - 0.713). The validation was performed in the test cohort with the C-index of 0.663 (95% CI, 0.646-0.680) on CSS and 0.651 (95% CI, 0.636 - 0.666). The calibration plots indicated an optimal agreement in the training cohort and satisfactory agreement in the test cohort between the nomogram prediction and the actual survival probability (**Figures 6**, **7**). The DCA indicated that using the nomograms for risk management can present positive clinical benefit when the threshold probability ranged from about 15% to 90%. The range of the threshold probability is covered with the death probability of the patients, which suggests the good clinical applicability of the nomograms. (**Figures 8**, **9**).

## Discussion

Approximately 85% of PCa patients suffer from bone metastasis which was reported as the most common metastasis type (12). It is closely related to mortality, negatively affecting the quality of life of PCa patients due to skeletal complications (13, 14). Advances in surgery, radiation, and chemotherapy have increased opportunities to diagnose and manage early-stage prostate cancer (15). However, there is no consensus currently on the clinical treatment selection protocols for the bone-metastatic PCa patients to extend their life expectancy (16).

A growing body of studies suggests that radical prostatectomy of the primary tumor can improve the survival outcomes with metastatic prostate cancer (17, 18). This fact was also borne out in randomized clinical trials in men with advanced PCa (19, 20). Chemotherapy treatment based on docetaxel has long been known to provide a survival advantage for patients with advanced prostate cancer after Petrylak et al. proved it in their two Phase III trials in 2004 (21). If patients are fit enough, chemotherapy was recommended with castration in the newly diagnosed M1 phase (22). In a study of 103 patients, more than 90% of which had symptomatic and progressive osseous metastases, Tu SM et al. confirmed chemotherapy effectively treated prostate cancer with the combination of radiopharmaceuticals (23).

In the present study, we respectively determined independent prognostic factors associated with overall and cancer-specific survival possibility of bone-metastatic PCa patients by using a large cohort of data retrieved from the SEER database with a long follow-up time. Consistent with previous studies, radical prostatectomy was found to be significantly associated with better CSS and OS. Focal therapy, on the other hand, was associated with an increased risk of overall and prostate cancer-specific mortality. This is probably because focal therapy could be utilized more ideally when patients have a low risk of cancer progression or metastasis (24). The potential for inadequate cancer control of focal therapy could lead to inferior outcomes due to inaccurate mapping of multifocal disease or suboptimal treatment performance (25). The finding of chemotherapy in this study was much the same as that reported previously, an effective way to increase overall survival among bone-metastatic PCa patients. It is worth pointing out that radiotherapy did not show any significant effect on CSS and OS. This can be explained by the dose and fractionation regimen of radiotherapy. Wallace et al. figured out from 569 bone-metastatic patients treated with radiotherapy that survival benefits would be counteracted for patients with poor prognosis by long-term radiation therapy (26).

Apart from the above factors, age and ethnicity were identified as predictors of survival for patients with prostate cancer (27, 28). More risks exist for mortality along with the raising age. Compared with the white, we found other races like American Indian/AK Native and Asian/Pacific Islander had a better survival probability, which matched the conclusions of previous research (29, 30). Marital status was formerly reported to associate with survival (31). Married patients were featured by better survival advantages compared with the single, probably due to definitive therapy and emotional support received (32). This was consistent with our study. Akoto et al. indicated a majority of PCa-related mortality results from metastatic disease that is characterized by metastasis of prostate tumor cells to various distant organs (33). Similarly, we identified patients with bone metastases had a higher risk of death if they had liver, lung, or brain metastases. With regard to Gleason score, PSA value, and clinic T/N stages, they were widely considered as the robust prognosis predictors and combined to analyze mortality risks of PCa patients in bone metastases (29, 34, 35). Multiple studies pointed out that bone-metastatic patients with a PSA level > 20ng/ml or a Gleason score >8 were considered for a higher lethal risk (36). Consistent with previous reports, PCa patients in bone metastases with higher PSA level or higher Gleason score were demonstrated with a worse survival probability.

Based on these prognostic factors, we established and tested two nomograms to predict the 3-year and 5-year OS and CSS of PCa patients with bone metastasis. Nomogram is a convenient approach to assist clinicians in predicting cancer risk and handling treatment strategies for individual patients (37, 38). The monogram for OS comprised age, race, marital status, surgery, chemotherapy, Gleason score, PSA value, tumor grade, T stage, liver, lung, and brain metastases. All the other factors except for brain metastases were included in building CSS nomogram. These nomograms were shown to have an ideal effect in predicting survival outcomes for individual bone-metastatic PCa patients by C-index, calibration curve, and DCA. Harrell C-index, the primary measure to evaluate the discrimination ability of nomograms, indicates a good match when values are over 0.7 (39). We respectively calculated the C-index for the CSS and OS model discrimination without risk factors as 0.502 (95% CI, 0.486-0.518) and 0.503 (95% CI, 0.486-0.521), while it showed a notable improvement in the prediction when adding the related prognostic factors in the nomograms (CSS model from 0.502 to 0.712 and OS model from 0.503 to 0.702). It is represented that these nomograms have efficiently involved the primary predictors that contribute to the survival outcomes.

Inevitably, this study has some limitations. Initially, this was a retrospective study, therefore, the potential risk of selection bias cannot be ruled out even after applying multivariate analysis and PSM. Secondly, as white people are predominantly recorded in the SEER database, it might partly limit the application of our nomograms in Asian patients. Thirdly, potential predictive factors such as radiation dose, specific chemotherapy regimens, and endocrine therapy were not considered in the nomograms due to lacking information from the SEER database, which could lead to the unoptimistic C-index in our invalidation model and further impact the evaluation of treatment results.

## Conclusion

This study provides a novel perspective on understanding the survival benefits of radical prostatectomy and chemotherapy for patients with bone metastasis. In addition, our nomograms provide the indicators and tools for prognostic evaluation among bone-metastatic PCa patients. However, these results need to be verified by further clinical studies.

## Data availability statement

Publicly available datasets were analyzed in this study. This data can be found here: Surveillance, Epidemiology, and End Results (SEER) database based on 17 registries across the United States between 2000 and 2019, submitted in Nov 2021, and released in April 2022.

## Author contributions

LW, ZJ and GQ conceptualized the study. DS, AL, ZS, SY, YS and AC participated in the data analysis. DS drafted the manuscript. LW, ZJ and GQ revised the final manuscript. All authors contributed to the article and approved the submitted version.
