# Supplementary material for: Nomograms predict survival benefits of radical prostatectomy and chemotherapy for prostate cancer with bone metastases: A SEER‐based study
Source: Front Oncol. 2022 Dec 6;12:1020898. doi: 10.3389/fonc.2022.1020898 (PMC9764338; doi:10.3389/fonc.2022.1020898)
Supplement: Supplementary file 1 [file DataSheet_1.docx]

| **Variables** | **Total Training Cohort** | **Chemotherapy No/Unknown** | **Chemotherapy Yes** | ***P*** |
| --- | --- | --- | --- | --- |
|  | **(n=5,120)** | **(n=4,298)** | **(n=822)** |  |
| **Age, n(%)** |  |  |  | <0.0001 |
| ≤ 73yrs | 3290 (64.3) | 2596 (60.4) | 694 (84.4) |  |
| 74-81yrs | 1066 (20.8) | 966 (22.5) | 100 (12.2) |  |
| > 81yrs | 764 (14.9) | 736 (17.1) | 28 (3.4) |  |
| **Race, n(%)** |  |  |  | 0.125 |
| White | 3925 (76.7) | 3281 (76.3) | 644 (78.3) |  |
| Black | 865 (16.9) | 727 (16.9) | 138 (16.8) |  |
| Others | 330 (6.4) | 290 (6.7) | 40 (4.9) |  |
| **Marital Status, n(%)** |  |  |  | 0.015 |
| Married | 3194 (62.4) | 2648 (61.6) | 546 (66.4) |  |
| Single | 891 (17.4) | 753 (17.5) | 138 (16.8) |  |
| Others | 1035 (20.2) | 897 (20.9) | 138 (16.8) |  |
| **Grade, n(%)** |  |  |  | 0.9013 |
| Low | 520 (10.2) | 438 (10.2) | 82 (10.0) |  |
| High | 4600 (89.8) | 3860 (89.8) | 740 (90.0) |  |
| **Gleason Score, n(%)** |  |  |  | <0.0001 |
| ≤ 7 | 870 (17.0) | 765 (17.8) | 105 (12.8) |  |
| 8 | 1244 (24.3) | 1065 (24.8) | 179 (21.8) |  |
| 9 | 2454 (47.9) | 2032 (47.3) | 422 (51.3) |  |
| 10 | 552 (10.8) | 436 (10.1) | 116 (14.1) |  |
| **PSA (ng/ml), n(%)** |  |  |  | 0.2803 |
| < 20 | 1259 (24.6) | 1071 (24.9) | 188 (22.9) |  |
| 20 - 50 | 936 (18.3) | 792 (18.4) | 144 (17.5) |  |
| ＞ 50 | 2925 (57.1) | 2435 (56.7) | 490 (59.6) |  |
| **Surgery, n(%)** |  |  |  | 0.0151 |
| No | 4409 (86.1) | 3675 (85.5) | 734 (89.3) |  |
| Focal Therapy | 572 (11.2) | 500 (11.6) | 72 (8.8) |  |
| Radical Prostatectomy | 139 (2.7) | 123 (2.9) | 16 (1.9) |  |
| **Radiation, n(%)** |  |  |  | 0.7496 |
| No/Unknown | 3912 (76.4) | 3288 (76.5) | 624 (75.9) |  |
| Yes | 1208 (23.6) | 1010 (23.5) | 198 (24.1) |  |
| **Brain Metastasis, n(%)** |  |  |  | 0.3008 |
| No | 5092 (99.5) | 4277 (99.5) | 815 (99.1) |  |
| Yes | 28 (0.5) | 21 (0.5) | 7 (0.9) |  |
| **Liver Metastasis, n(%)** |  |  |  | <0.0001 |
| No | 4969 (97.1) | 4199 (97.7) | 770 (93.7) |  |
| Yes | 151 (2.9) | 99 (2.3) | 52 (6.3) |  |
| **Lung Metastasis, n(%)** |  |  |  | <0.0001 |
| No | 4841 (94.6) | 4097 (95.3) | 744 (90.5) |  |
| Yes | 279 (5.4) | 201 (4.7) | 78 (9.5) |  |
| **T Stage, n(%)** |  |  |  | 0.0607 |
| T1/T2 | 4454 (87.0) | 3756 (87.4) | 698 (84.9) |  |
| T3/T4 | 666 (13.0) | 542 (12.6) | 124 (15.1) |  |
| **N Stage, n(%)** |  |  |  | <0.0001 |
| N0 | 3532 (69.0) | 3058 (71.1) | 474 (57.7) |  |
| N1 | 1588 (31.0) | 1240 (28.9) | 348 (42.3) |  |

Supplementary Table 1. Baseline characteristics of the 5,120 bone-metastatic PCa patients in the training cohort by chemotherapy stratification.

| **Variables** | **Total Training Cohort** | **Radical Prostatectomy No** | **Radical Prostatectomy Yes** | ***P*** |
| --- | --- | --- | --- | --- |
|  | **(n=5,120)** | **(n=4,981)** | **(n=139)** |  |
| **Age, n(%)** |  |  |  | <0.0001 |
| ≤ 73yrs | 3290 (64.3) | 3162 (63.5) | 128 (92.1) |  |
| 74-81yrs | 1066 (20.8) | 1056 (21.2) | 10 (7.2) |  |
| > 81yrs | 764 (14.9) | 763 (15.3) | 1 (0.7) |  |
| **Race, n(%)** |  |  |  | 0.3851 |
| White | 3925 (76.7) | 3815 (76.6) | 110 (79.1) |  |
| Black | 865 (16.9) | 847 (17.0) | 18 (12.9) |  |
| Others | 330 (6.4) | 319 (6.4) | 11 (7.9) |  |
| **Marital Status, n(%)** |  |  |  | 0.0001 |
| Married | 3194 (62.4) | 3083 (61.9) | 111 (79.9) |  |
| Single | 891 (17.4) | 876 (17.6) | 15 (10.8) |  |
| Others | 1035 (20.2) | 1022 (20.5) | 13 (9.4) |  |
| **Grade, n(%)** |  |  |  | 0.0076 |
| Low | 520 (10.2) | 496 (10.0) | 24 (17.3) |  |
| High | 4600 (89.8) | 4485 (90.0) | 115 (82.7) |  |
| **Gleason Score, n(%)** |  |  |  | <0.0001 |
| ≤ 7 | 870 (17.0) | 813 (16.3) | 57 (41.0) |  |
| 8 | 1244 (24.3) | 1201 (24.1) | 43 (30.9) |  |
| 9 | 2454 (47.9) | 2418 (48.5) | 36 (25.9) |  |
| 10 | 552 (10.8) | 549 (11.0) | 3 (2.2) |  |
| **PSA (ng/ml), n(%)** |  |  |  | <0.0001 |
| < 20 | 1259 (24.6) | 1161 (23.3) | 98 (70.5) |  |
| 20 - 50 | 936 (18.3) | 914 (18.3) | 22 (15.8) |  |
| ＞ 50 | 2925 (57.1) | 2906 (58.3) | 19 (13.7) |  |
| **Radiation, n(%)** |  |  |  | 0.1395 |
| No/Unknown | 3912 (76.4) | 3798 (76.2) | 114 (82.0) |  |
| Yes | 1208 (23.6) | 1183 (23.8) | 25 (18.0) |  |
| **Chemotherapy, n(%)** |  |  |  | 0.1731 |
| No/Unknown | 4298 (83.9) | 4175 (83.8) | 123 (88.5) |  |
| Yes | 822 (16.1) | 806 (16.2) | 16 (11.5) |  |
| **Brain Metastasis, n(%)** |  |  |  | 1 |
| No | 5092 (99.5) | 4954 (99.5) | 138 (99.3) |  |
| Yes | 28 (0.5) | 27 (0.5) | 1 (0.7) |  |
| **Liver Metastasis, n(%)** |  |  |  | 0.4162 |
| No | 4969 (97.1) | 4832 (97.0) | 137 (98.6) |  |
| Yes | 151 (2.9) | 149 (3.0) | 2 (1.4) |  |
| **Lung Metastasis, n(%)** |  |  |  | 0.2441 |
| No | 4841 (94.6) | 4706 (94.5) | 135 (97.1) |  |
| Yes | 279 (5.4) | 275 (5.5) | 4 (2.9) |  |
| **T Stage, n(%)** |  |  |  | 0.0143 |
| T1/T2 | 4454 (87.0) | 4323 (86.8) | 131 (94.2) |  |
| T3/T4 | 666 (13.0) | 658 (13.2) | 8 (5.8) |  |
| **N Stage, n(%)** |  |  |  | 0.0809 |
| N0 | 3532 (69.0) | 3446 (69.2) | 86 (61.9) |  |
| N1 | 1588 (31.0) | 1535 (30.8) | 53 (38.1) |  |

Supplementary Table 2. Baseline characteristics of the 5,120 bone-metastatic PCa patients in the training cohort by radical prostatectomy stratification.

| **Variables** | **Total Training Cohort** | **Chemotherapy No/Unknown** | **Chemotherapy Yes** | ***P*** |
| --- | --- | --- | --- | --- |
|  | **(n=1,644)** | **(n=822)** | **(n=822)** |  |
| **Age, n(%)** |  |  |  | 0.903 |
| ≤ 73yrs | 1391 (84.6) | 697 (84.8) | 694 (84.4) |  |
| 74-81yrs | 195 (11.9) | 95 (11.6) | 100 (12.2) |  |
| > 81yrs | 58 (3.5) | 30 (3.6) | 28 (3.4) |  |
| **Race, n(%)** |  |  |  | 0.125 |
| White | 1253 (76.2) | 609 (74.1) | 644 (78.3) |  |
| Black | 305 (18.6) | 167 (20.3) | 138 (16.8) |  |
| Others | 86 (5.2) | 46 (5.6) | 40 (4.9) |  |
| **Marital Status, n(%)** |  |  |  | 0.877 |
| Married | 1083 (65.9) | 537 (65.3) | 546 (66.4) |  |
| Single | 283 (17.2) | 145 (17.6) | 138 (16.8) |  |
| Others | 278 (16.9) | 140 (17.0) | 138 (16.8) |  |
| **Grade, n(%)** |  |  |  | 0.803 |
| Low | 160 (9.7) | 78 (9.5) | 82 (10.0) |  |
| High | 1484 (90.3) | 744 (90.5) | 740 (90.0) |  |
| **Gleason Score, n(%)** |  |  |  | 0.960 |
| ≤ 7 | 203 (12.3) | 98 (11.9) | 105 (12.8) |  |
| 8 | 358 (21.8) | 179 (21.8) | 179 (21.8) |  |
| 9 | 848 (51.6) | 426 (51.8) | 422 (51.3) |  |
| 10 | 235 (14.3) | 119 (14.5) | 116 (14.1) |  |
| **PSA (ng/ml), n(%)** |  |  |  | 0.861 |
| < 20 | 368 (22.4) | 180 (21.9) | 188 (22.9) |  |
| 20 - 50 | 294 (17.9) | 150 (18.2) | 144 (17.5) |  |
| ＞ 50 | 982 (59.7) | 492 (59.9) | 490 (59.6) |  |
| **Surgery, n(%)** |  |  |  | 0.967 |
| No | 1471 (89.5) | 737 (89.7) | 734 (89.3) |  |
| Focal Therapy | 142 (8.6) | 70 (8.5) | 72 (8.8) |  |
| Radical Prostatectomy | 31 (1.9) | 15 (1.8) | 16 (1.9) |  |
| **Radiation, n(%)** |  |  |  | 1.000 |
| No/Unknown | 1249 (76.0) | 625 (76.0) | 624 (75.9) |  |
| Yes | 395 (24.0) | 197 (24.0) | 198 (24.1) |  |
| **Brain Metastasis, n(%)** |  |  |  | 0.545 |
| No | 1633 (99.3) | 818 (99.5) | 815 (99.1) |  |
| Yes | 11 (0.7) | 4 (0.5) | 7 (0.9) |  |
| **Liver Metastasis, n(%)** |  |  |  | 0.602 |
| No | 1546 (94.0) | 776 (94.4) | 770 (93.7) |  |
| Yes | 98 (6.0) | 46 (5.6) | 52 (6.3) |  |
| **Lung Metastasis, n(%)** |  |  |  | 0.668 |
| No | 1494 (90.9) | 750 (91.2) | 744 (90.5) |  |
| Yes | 150 (9.1) | 72 (8.8) | 78 (9.5) |  |
| **T Stage, n(%)** |  |  |  | 0.587 |
| T1/T2 | 1387 (84.4) | 689 (83.8) | 698 (84.9) |  |
| T3/T4 | 257 (15.6) | 133 (16.2) | 124 (15.1) |  |
| **N Stage, n(%)** |  |  |  | 0.727 |
| N0 | 940 (57.2) | 466 (56.7) | 474 (57.7) |  |
| N1 | 704 (42.8) | 356 (43.3) | 348 (42.3) |  |

Supplementary Table 3. Baseline characteristics of patients in 1:1 matched group by PSM on chemotherapy stratification

| **Variables** | **Total Training Cohort** | **Radical Prostatectomy No/Unknown** | **Radical Prostatectomy Yes** | ***P*** |
| --- | --- | --- | --- | --- |
|  | **(n=278)** | **(n=139)** | **(n=139)** |  |
| **Age, n(%)** |  |  |  | 1.000 |
| ≤ 73yrs | 256 (92.1) | 128 (92.1) | 128 (92.1) |  |
| 74-81yrs | 2 (0.7) | 1 (0.7) | 1 (0.7) |  |
| > 81yrs | 20 (7.2) | 10 (7.2) | 10 (7.2) |  |
| **Race, n(%)** |  |  |  | 0.645 |
| White | 219 (78.8) | 109 (78.4) | 110 (79.1) |  |
| Black | 40 (14.4) | 22 (15.8) | 18 (12.9) |  |
| Others | 19 (6.8) | 8 (5.8) | 11 (7.9) |  |
| **Marital Status, n(%)** |  |  |  | 0.923 |
| Married | 224 (80.6) | 113 (81.3) | 111 (79.9) |  |
| Single | 28 (10.1) | 13 (9.4) | 15 (10.8) |  |
| Others | 26 (9.4) | 13 (9.4) | 13 (9.4) |  |
| **Grade, n(%)** |  |  |  | 1.000 |
| Low | 47 (16.9) | 23 (16.5) | 24 (17.3) |  |
| High | 231 (83.1) | 116 (83.5) | 115 (82.7) |  |
| **Gleason Score, n(%)** |  |  |  | 0.938 |
| ≤ 7 | 111 (39.9) | 54 (38.8) | 57 (41.0) |  |
| 8 | 91 (32.7) | 48 (34.5) | 43 (30.9) |  |
| 9 | 70 (25.2) | 34 (24.5) | 36 (25.9) |  |
| 10 | 6 (2.2) | 3 (2.2) | 3 (2.2) |  |
| **PSA (ng/ml), n(%)** |  |  |  | 0.986 |
| < 20 | 197 (70.9) | 99 (71.2) | 98 (70.5) |  |
| 20 - 50 | 43 (15.5) | 21 (15.1) | 22 (15.8) |  |
| ＞ 50 | 38 (13.7) | 19 (13.7) | 19 (13.7) |  |
| **Chemotherapy, n(%)** |  |  |  | 0.855 |
| No/Unknown | 244 (87.8) | 121 (87.1) | 123 (88.5) |  |
| Yes | 34 (12.2) | 18 (12.9) | 16 (11.5) |  |
| **Radiation, n(%)** |  |  |  | 1.000 |
| No/Unknown | 229 (82.4) | 115 (82.7) | 114 (82.0) |  |
| Yes | 49 (17.6) | 24 (17.3) | 25 (18.0) |  |
| **Brain Metastasis, n(%)** |  |  |  | 1.000 |
| No | 277 (99.6) | 139 (100.0) | 138 (99.3) |  |
| Yes | 1 (0.4) | 0 (0.0) | 1 (0.7) |  |
| **Liver Metastasis, n(%)** |  |  |  | 1.000 |
| No | 274 (98.6) | 137 (98.6) | 137 (98.6) |  |
| Yes | 4 (1.4) | 2 (1.4) | 2 (1.4) |  |
| **Lung Metastasis, n(%)** |  |  |  | 1.000 |
| No | 270 (97.1) | 135 (97.1) | 135 (97.1) |  |
| Yes | 8 (2.9) | 4 (2.9) | 4 (2.9) |  |
| **T Stage, n(%)** |  |  |  | 1.000 |
| T1/T2 | 262 (94.2) | 131 (94.2) | 131 (94.2) |  |
| T3/T4 | 16 (5.8) | 8 (5.8) | 8 (5.8) |  |
| **N Stage, n(%)** |  |  |  | 0.901 |
| N0 | 174 (62.6) | 88 (63.3) | 86 (61.9) |  |
| N1 | 104 (37.4) | 51 (36.7) | 53 (38.1) |  |

Supplementary Table 4. Baseline characteristics of patients in 1:1 matched group by PSM on radical prostatectomy stratification

| **Variables** | **Prostate Cancer-Specific Survival** | | | | |  | **Overall Survival** | | | | |
| --- | --- | --- | --- | --- | --- | --- | --- | --- | --- | --- | --- |
|  | **Univariate Analysis** | |  | **Multivariate analysis** | |  | **Univariate Analysis** | |  | **Multivariate analysis** | |
|  | **HR (95% CI）** | ***P*** |  | **HR (95% CI）** | ***P*** |  | **HR (95% CI）** | ***P*** |  | **HR (95% CI）** | ***P*** |
| **Age** |  |  |  |  |  |  |  |  |  |  |  |
| ≤ 73yrs | Reference |  |  | Reference |  |  | Reference |  |  | Reference |  |
| 74-81yrs | **1.31 (1.08-1.58)** | **0.006** |  | 1.22 (1.00 - 1.48) | 0.051 |  | **1.37 (1.14-1.63)** | **0.001** |  | **1.28 (1.07 - 1.54)** | **0.007** |
| ＞ 81yrs | **1.87 (1.37-2.56)** | **< 0.001** |  | **1.59 (1.15 - 2.18)** | **0.005** |  | **2.03 (1.53-2.7)** | **< 0.001** |  | **1.68 (1.25 - 2.24)** | **< 0.001** |
| **Race** |  |  |  |  |  |  |  |  |  |  |  |
| White | Reference |  |  | Reference |  |  | Reference |  |  | Reference |  |
| Black | 1.03 (0.87-1.21) | 0.747 |  | 0.98 (0.82 - 1.16) | 0.790 |  | 1.07 (0.92-1.24) | 0.403 |  | 1.02 (0.87 - 1.19) | 0.807 |
| Others | **0.6 (0.43-0.83)** | **0.002** |  | **0.61 (0.44 - 0.85)** | **0.004** |  | **0.6 (0.44-0.82)** | **0.001** |  | **0.61 (0.44 - 0.83)** | **0.002** |
| **Marital Status** |  |  |  |  |  |  |  |  |  |  |  |
| Married | Reference |  |  | Reference |  |  | Reference |  |  | Reference |  |
| Single | 1.17 (0.99-1.39) | 0.071 |  | 1.06 (0.88 - 1.26) | 0.550 |  | **1.18 (1-1.38)** | **0.049** |  | 1.06 (0.90 - 1.26) | 0.481 |
| Others | 1.15 (0.97-1.36) | 0.12 |  | 1.03 (0.87 - 1.23) | 0.710 |  | **1.23 (1.05-1.44)** | **0.012** |  | 1.12 (0.95 - 1.32) | 0.176 |
| **Grade** |  |  |  |  |  |  |  |  |  |  |  |
| Low | Reference |  |  | Reference |  |  | Reference |  |  | Reference |  |
| High | **1.86 (1.44-2.41)** | **< 0.001** |  | **2.20 (1.40 - 3.45)** | **< 0.001** |  | **1.88 (1.47-2.39)** | **< 0.001** |  | **2.69 (1.80 - 4.01)** | **< 0.001** |
| **Gleason Score** |  |  |  |  |  |  |  |  |  |  |  |
| ≤ 7 | Reference |  |  | Reference |  |  | Reference |  |  | Reference |  |
| 8 | 0.95 (0.73-1.23) | 0.689 |  | **0.48 (0.32 - 0.72)** | **< 0.001** |  | 0.93 (0.74-1.18) | 0.564 |  | **0.42 (0.30 - 0.60)** | **< 0.001** |
| 9 | **1.75 (1.4-2.19)** | **< 0.001** |  | 0.89 (0.61 - 1.31) | 0.561 |  | **1.64 (1.33-2.01)** | **< 0.001** |  | 0.73 (0.52 - 1.02) | 0.065 |
| 10 | **2.6 (2.02-3.35)** | **< 0.001** |  | 1.24 (0.82 - 1.87) | 0.303 |  | **2.41 (1.9-3.05)** | **< 0.001** |  | 1.00 (0.70 - 1.43) | 0.984 |
| **PSA (ng/ml)** |  |  |  |  |  |  |  |  |  |  |  |
| < 20 | Reference |  |  | Reference |  |  | Reference |  |  | Reference |  |
| 20 - 50 | **1.26 (1.02-1.55)** | **0.028** |  | 1.07 (0.87 - 1.31) | 0.550 |  | **1.34 (1.1-1.62)** | **0.003** |  | 1.14 (0.94 - 1.38) | 0.194 |
| ＞ 50 | **1.39 (1.18-1.64)** | **< 0.001** |  | 1.15 (0.97 - 1.36) | 0.105 |  | **1.46 (1.25-1.7)** | **< 0.001** |  | **1.20 (1.02 - 1.41)** | **0.024** |
| **Surgery** |  |  |  |  |  |  |  |  |  |  |  |
| No | Reference |  |  | Reference |  |  | Reference |  |  | Reference |  |
| Focal Therapy | 1.76 (1.44-2.15) | **< 0.001** |  | **1.50 (1.22 - 1.85)** | **< 0.001** |  | **1.72 (1.42-2.08)** | **< 0.001** |  | **1.48 (1.22 - 1.80)** | **< 0.001** |
| Radical Prostatectomy | **0.31 (0.16-0.63)** | **0.001** |  | **0.37 (0.18 - 0.76)** | **0.007** |  | **0.27 (0.14-0.55)** | **< 0.001** |  | **0.32 (0.16 - 0.65)** | **0.002** |
| **Radiation** |  |  |  |  |  |  |  |  |  |  |  |
| No/Unknown | Reference |  |  | Reference |  |  | Reference |  |  | Reference |  |
| Yes | **1.18 (1.02-1.36)** | **0.024** |  | **1.17 (1.01 - 1.35)** | **0.039** |  | **1.15 (1-1.32)** | **0.045** |  | 1.13 (0.99 - 1.30) | 0.076 |
| **Chemotherapy** |  |  |  |  |  |  |  |  |  |  |  |
| No/Unknown | Reference |  |  | Reference |  |  | Reference |  |  | Reference |  |
| Yes | 0.9 (0.79-1.02) | 0.094 |  | **0.87 (0.77 - 0.99)** | **0.039** |  | **0.85 (0.75-0.95)** | **0.007** |  | **0.83 (0.73 - 0.93)** | **0.002** |
| **Brain Metastasis** |  |  |  |  |  |  |  |  |  |  |  |
| No | Reference |  |  | Reference |  |  | Reference |  |  | Reference |  |
| Yes | 1.65 (0.86-3.19) | 0.134 |  | 0.71 (0.35 - 1.43) | 0.337 |  | 1.45 (0.75-2.79) | 0.269 |  | 0.66 (0.33 - 1.32) | 0.243 |
| **Liver Metastasis** |  |  |  |  |  |  |  |  |  |  |  |
| No | Reference |  |  | Reference |  |  | Reference |  |  | Reference |  |
| Yes | **2.85 (2.27-3.57)** | **< 0.001** |  | **2.49 (1.95 - 3.19)** | **< 0.001** |  | **2.66 (2.14-3.31)** | **< 0.001** |  | **2.38 (1.87 - 3.02)** | **< 0.001** |
| **Lung Metastasis** |  |  |  |  |  |  |  |  |  |  |  |
| No | Reference |  |  | Reference |  |  | Reference |  |  | Reference |  |
| Yes | **1.51 (1.23-1.85)** | **< 0.001** |  | **1.48 (1.18 - 1.85)** | **< 0.001** |  | **1.44 (1.19-1.75)** | **< 0.001** |  | **1.42 (1.15 - 1.76)** | **0.001** |
| **T Stage** |  |  |  |  |  |  |  |  |  |  |  |
| T1/T2 | Reference |  |  | Reference |  |  | Reference |  |  | Reference |  |
| T3/T4 | **1.41 (1.2-1.66)** | **< 0.001** |  | **1.20 (1.01 - 1.43)** | **0.034** |  | **1.36 (1.16-1.59)** | **< 0.001** |  | 1.17 (1.00 - 1.38) | 0.053 |
| **N Stage** |  |  |  |  |  |  |  |  |  |  |  |
| N0 | Reference |  |  | Reference |  |  | Reference |  |  | Reference |  |
| N1 | 1.12 (0.99-1.27) | 0.083 |  | 1.05 (0.92 - 1.20) | 0.466 |  | 1.06 (0.94-1.2) | 0.333 |  | 1.01 (0.89 - 1.14) | 0.903 |

Supplementary Table 5. Univariate and multivariate analysis of CSS and OS after PSM on chemotherapy stratification

| **Variables** | **Prostate Cancer-Specific Survival** | | | | |  | **Overall Survival** | | | | |
| --- | --- | --- | --- | --- | --- | --- | --- | --- | --- | --- | --- |
|  | **Univariate Analysis** | |  | **Multivariate analysis** | |  | **Univariate Analysis** | |  | **Multivariate analysis** | |
|  | **HR (95% CI）** | ***P*** |  | **HR (95% CI）** | ***P*** |  | **HR (95% CI）** | ***P*** |  | **HR (95% CI）** | ***P*** |
| **Age** |  |  |  |  |  |  |  |  |  |  |  |
| ≤ 73yrs | Reference |  |  | Reference |  |  | Reference |  |  | Reference |  |
| 74-81yrs | **2.25 (1.2-4.24)** | **0.012** |  | 1.4(0.58 - 3.38) | 0.457 |  | **2.33 (1.33-4.08)** | **0.003** |  | 1.27(0.59 - 2.74) | 0.536 |
| ＞ 81yrs | 4.84 (0.66-35.41) | 0.12 |  | 0.14(0.01 - 1.99) | 0.146 |  | **8.32 (2.01-34.52)** | **0.003** |  | 0.35(0.04 - 3.08) | 0.347 |
| **Race** |  |  |  |  |  |  |  |  |  |  |  |
| White | Reference |  |  | Reference |  |  | Reference |  |  | Reference |  |
| Black | **1.7 (1.03-2.8)** | **0.038** |  | 1.68(0.95 - 2.96) | 0.073 |  | **1.68 (1.07-2.63)** | **0.023** |  | 1.64(1.00 - 2.69) | 0.052 |
| Others | 0.64 (0.26-1.6) | 0.345 |  | 0.63(0.25 - 1.62) | 0.341 |  | 0.63 (0.27-1.43) | 0.267 |  | 0.61(0.26 - 1.43) | 0.253 |
| **Marital Status** |  |  |  |  |  |  |  |  |  |  |  |
| Married | Reference |  |  | Reference |  |  | Reference |  |  | Reference |  |
| Single | 1.76 (0.95-3.24) | 0.072 |  | 1.08(0.5 - 2.33) | 0.837 |  | 1.54 (0.86-2.76) | 0.149 |  | 0.91(0.44 - 1.88) | 0.803 |
| Others | 1.51 (0.78-2.94) | 0.222 |  | **2.63(1.23 - 5.62)** | **0.012** |  | **1.69 (0.96-2.98)** | **0.067** |  | **2.66(1.38 - 5.11)** | **0.003** |
| **Grade** |  |  |  |  |  |  |  |  |  |  |  |
| Low | Reference |  |  | Reference |  |  | Reference |  |  | Reference |  |
| High | **2.18 (1.06-4.51)** | **0.035** |  | 1.24(0.53 - 2.92) | 0.624 |  | 1.62 (0.91-2.88) | 0.103 |  | 0.95(0.47 - 1.9) | 0.883 |
| **Gleason Score** |  |  |  |  |  |  |  |  |  |  |  |
| ≤ 7 | Reference |  |  | Reference |  |  | Reference |  |  | Reference |  |
| 8 | 1.03 (0.6-1.76) | 0.927 |  | 0.83(0.45 - 1.54) | 0.558 |  | 1.05 (0.66-1.67) | 0.84 |  | 0.95(0.55 - 1.62) | 0.846 |
| 9 | **2.64 (1.62-4.32)** | **< 0.001** |  | **2.12(1.13 - 3.98)** | **0.019** |  | **2.2 (1.41-3.44)** | **0.001** |  | **2.01(1.14 - 3.54)** | **0.016** |
| 10 | **5.37 (1.88-15.33)** | **0.002** |  | 2.68(0.61 - 11.72) | 0.191 |  | **5.24 (2.05-13.35)** | **0.001** |  | 2.62(0.64 - 10.77) | 0.182 |
| **PSA (ng/ml)** |  |  |  |  |  |  |  |  |  |  |  |
| < 20 | Reference |  |  | Reference |  |  | Reference |  |  | Reference |  |
| 20 - 50 | **1.94 (1.15-3.28)** | **0.013** |  | **2.25(1.21 - 4.18)** | **0.011** |  | **1.76 (1.09-2.85)** | **0.022** |  | **2.21(1.27 - 3.85)** | **0.005** |
| ＞ 50 | **3.23 (1.94-5.38)** | **< 0.001** |  | **3.32(1.71 - 6.44)** | **< 0.001** |  | **3.16 (2-4.99)** | **<0.001** |  | **3.34(1.84 - 6.07)** | **<0.001** |
| **Surgery** |  |  |  |  |  |  |  |  |  |  |  |
| No | Reference |  |  | Reference |  |  | Reference |  |  | Reference |  |
| Focal Therapy | **2.57 (1.27-5.23)** | **0.009** |  | **5.7(2.47 - 13.12)** | **< 0.001** |  | **2.37 (1.22-4.63)** | **0.011** |  | **3.64(1.67 - 7.93)** | **0.001** |
| Radical Prostatectomy | **0.32 (0.2-0.51)** | **< 0.001** |  | **0.22(0.14 - 0.36)** | **< 0.001** |  | **0.35 (0.23-0.52)** | **<0.001** |  | **0.26(0.17 - 0.41)** | **<0.001** |
| **Radiation** |  |  |  |  |  |  |  |  |  |  |  |
| No/Unknown | Reference |  |  | Reference |  |  | Reference |  |  | Reference |  |
| Yes | 1.07 (0.63-1.82) | 0.79 |  | 1.01(0.56 - 1.82) | 0.979 |  | 1.00 (0.62-1.62) | 0.994 |  | 0.94(0.55 - 1.61) | 0.823 |
| **Chemotherapy** |  |  |  |  |  |  |  |  |  |  |  |
| No/Unknown | Reference |  |  | Reference |  |  | Reference |  |  | Reference |  |
| Yes | 1.13 (0.6-2.12) | 0.71 |  | 0.59(0.29 - 1.21) | 0.152 |  | 0.89 (0.48-1.66) | 0.707 |  | 0.54(0.27 - 1.07) | 0.078 |
| **Liver Metastasis** |  |  |  |  |  |  |  |  |  |  |  |
| No | Reference |  |  | Reference |  |  | Reference |  |  | Reference |  |
| Yes | **3.71 (1.17-11.77)** | **0.026** |  | **4.82(1.28 - 18.14)** | **0.020** |  | 3 (0.95-9.49) | 0.061 |  | **4.07(1.12 - 14.75)** | **0.033** |
| **Lung Metastasis** |  |  |  |  |  |  |  |  |  |  |  |
| No | Reference |  |  | Reference |  |  | Reference |  |  | Reference |  |
| Yes | **3.64 (1.59-8.36)** | **0.002** |  | **5.53(2.01 - 15.24)** | **< 0.001** |  | **3.48 (1.61-7.5)** | **0.001** |  | **4.91(1.97 - 12.25)** | **0.001** |
| **T Stage** |  |  |  |  |  |  |  |  |  |  |  |
| T1/T2 | Reference |  |  | Reference |  |  | Reference |  |  | Reference |  |
| T3/T4 | **4.05 (2.15-7.62)** | **< 0.001** |  | **5.72(2.61 - 12.52)** | **< 0.001** |  | **3.24 (1.74-6.06)** | **<0.001** |  | **4.34(2.03 - 9.28)** | **<0.001** |
| **N Stage** |  |  |  |  |  |  |  |  |  |  |  |
| N0 | Reference |  |  | Reference |  |  | Reference |  |  | Reference |  |
| N1 | **2.05 (1.36-3.09)** | **0.001** |  | **2.15(1.26 - 3.67)** | **0.005** |  | **1.54 (1.06-2.23)** | **0.023** |  | 1.55(0.96 - 2.49) | 0.071 |

Supplementary Table 6. Univariate and multivariate analysis of CSS and OS after PSM on radical prostatectomy stratification
