# Supplementary material for: Nomograms predict survival benefits of radical prostatectomy and chemotherapy for prostate cancer with bone metastases: A SEER‐based study
Source: Front Oncol. 2022 Dec 6;12:1020898. doi: 10.3389/fonc.2022.1020898 (PMC9764338; doi:10.3389/fonc.2022.1020898)
Supplement: Supplementary file 2 [file DataSheet_2.docx]

The crucial codes are as follows:

library(tableone)

library(plyr)

library(survival)

library(survminer)

library(MatchIt)

library(ggDCA)

library(dcurves)

library(foreign)

library(tidyr)

library(dplyr)

library(rms)

library(pec)

library(prodlim)

#######################

vars <- c("Age","Race","Surgery","Marital", "Radiation","Chemotherapy","brainMeta","liverMeta",

"lungMeta","Grade", "Gleason","PSA", "TStage","N")

catvars <- c("Age","Race","Surgery","Marital", "Radiation","Chemotherapy","brainMeta","liverMeta",

"lungMeta","Grade", "Gleason","PSA", "TStage","N")

tableone <- CreateTableOne(vars = vars,factorVars = catvars,strata="cohort",data = data)

table1 <- print(tableone, contdigits=3,pDigits =4,showAllLevels = TRUE, quote = FALSE, noSpace = TRUE, printToggle = FALSE)

write.csv(table1,"table1.csv")

tableall<- CreateTableOne(vars = vars,factorVars = catvars,data = data)

table0 <- print(tableall, contdigits=3,pDigits =4,showAllLevels = TRUE, quote = FALSE, noSpace = TRUE, printToggle = FALSE)

########Univariate COX regression#####################

y<- Surv(time = aa$Survival,event = aa$CSS==0)

Uni_cox_model<- function(x){

FML <- as.formula(paste0 ("y~",x))

cox<- coxph(FML,data=aa)

cox1<-summary(cox)

HR <- round(cox1$coefficients[,2],2)

PValue <- round(cox1$coefficients[,5],3)

CI5 <-round(cox1$conf.int[,3],2)

CI95 <-round(cox1$conf.int[,4],2)

Uni_cox_model<- data.frame(

names <-rownames(cox1$conf.int),

'HR' = HR,

'CI5' = CI5,

'CI95' = CI95,

'P' = PValue)

return(Uni_cox_model)

}

names(aa)

variable.names<- colnames(aa)[c(1,3:4,12:18,20:21,24:26)];variable.names

Uni_cox <- lapply(variable.names, Uni_cox_model)

Uni_cox<- ldply(Uni_cox, data.frame)

Uni_cox$HR.CI95<-paste0(Uni_cox$HR," (",Uni_cox$CI5,'-',Uni_cox$CI95,")");Uni_cox

write.csv(Uni_cox)

yos<- Surv(time = aa$Survival,event = aa$OS==0)

Uni_cox_modelos<- function(x){

FMLos <- as.formula(paste0 ("yos~",x))

coxos<- coxph(FMLos,data=aa)

cox1os<-summary(coxos)

HR <- round(cox1os$coefficients[,2],2)

PValue <- round(cox1os$coefficients[,5],3)

CI5 <-round(cox1os$conf.int[,3],2)

CI95 <-round(cox1os$conf.int[,4],2)

Uni_cox_modelos<- data.frame(

names <-rownames(cox1os$conf.int),

'HR' = HR,

'CI5' = CI5,

'CI95' = CI95,

'P' = PValue)

return(Uni_cox_modelos)

}

variable.names<- colnames(aa)[c(1,3:4,12:18,20:21,24:26)];variable.names

Uni_coxos <- lapply(variable.names, Uni_cox_modelos)

Uni_coxos<- ldply(Uni_coxos, data.frame)

Uni_coxos$HR.CI95<-paste0(Uni_coxos$HR," (",Uni_coxos$CI5,'-',Uni_coxos$CI95,")");Uni_coxos

write.csv(Uni_coxos)

#######Multivariate COX regression#######

mul_cox<-coxph(Surv(time = aa$Survival,event = aa$CSS==0)~ Age+Race+Marital+Surgery+Radiation+Chemotherapy+Grade+Gleason+PSA+TStage+N+liverMeta+lungMeta+brainMeta,data=aa)

summary(mul_cox)

coxcss1<-summary(mul_cox)

coxcss1$coefficients

coxcss1$conf.int

mul_HR<- round(coxcss1$coefficients[,2],2)

mul_PValue<- round(coxcss1$coefficients[,5],4)

mul_CI1<-round(coxcss1$conf.int[,3],2)

mul_CI2<-round(coxcss1$conf.int[,4],2)

mul_CI95<-paste(mul_CI1,'-',mul_CI2)

mul_coxcss1 <- data.frame("HR" =mul_HR,

"CI95" =mul_CI95,

"P"=mul_PValue);mul_coxcss1

mul_cox3<-coxph(Surv(time = aa$Survival,event = aa$OS==0)~ Age+Race+Marital+Surgery+Grade+Gleason+Radiation+Chemotherapy+PSA+TStage+N+liverMeta+lungMeta+brainMeta,data=aa)

summary(mul_cox3)

coxos<-summary(mul_cox3)

coxos$coefficients

coxos$conf.int

mul_HR<- round(coxos$coefficients[,2],2)

mul_PValue<- round(coxos$coefficients[,5],4)

mul_CI1<-round(coxos$conf.int[,3],2)

mul_CI2<-round(coxos$conf.int[,4],2)

mul_CI95<-paste(mul_CI1,'-',mul_CI2)

mul_coxos <- data.frame("HR" =mul_HR,

"CI95" =mul_CI95,

"P"=mul_PValue);mul_coxos

####K-M survival curves using age, surgery and chemotherapy for examples#######

kmcssage<-survfit(Surv(time = aa$Survival,event =aa$CSS==0)~Age,data=aa)

summary(kmcssage)

summary(kmcssage,time=c(36,60))

ggsurvplot(kmcssage,

pval=TRUE,

pval.coord = c(0, 0.4),

xlab="Survival Months",

ylab="Cancer Specific Survival",

legend= "left",

break.x.by=12,

xlim=c(0,96),

pval.size =5,

risk.table=TRUE,

risk.table.height = 0.25,

palette="lancet",

title="CSS (n=5,120)",

legend.labs=c("＞81yrs","≤73yrs","74-81yrs"),

legend.title="Age",

surv.median.line = "hv")

kmosage<-survfit(Surv(time = aa$Survival,event =aa$OS==0)~Age,data=aa)

summary(kmosage)

summary(kmosage,time=c(36,60))

ggsurvplot(kmosage,

pval=TRUE,

pval.coord = c(0, 0.4),

xlab="Survival Months",

ylab="Overall Survival",

legend= "left",

break.x.by=12,

xlim=c(0,96),

pval.size =5,

risk.table=TRUE,

risk.table.height = 0.25,

palette="lancet",

title="OS (n=5,120)",

legend.labs=c("＞81yrs","≤73yrs","74-81yrs"),

legend.title="Age",

surv.median.line = "hv")

kmcss1<-survfit(Surv(time = aa$Survival,event =aa$CSS==0)~Surgery,data=aa)

summary(kmcss1)

summary(kmcss1,time=c(36,60))

ggsurvplot(kmcss1,

pval=TRUE,

pval.coord = c(0, 0.4),

xlab="Survival Months",

ylab="Cancer Specific Survival",

legend= "left",

break.x.by=12,

xlim=c(0,96),

pval.size =5,

risk.table=TRUE,

risk.table.height = 0.25,

palette="lancet",

title="CSS (n=5,120)",

legend.labs=c("No","Local Surgery","Radical Surgery"),

legend.title="Surgery",

surv.median.line = "hv")

kmos1<-survfit(Surv(time = aa$Survival,event =aa$OS==0)~Surgery,data=aa)

summary(kmos1)

summary(kmos1,time=c(36,60))

ggsurvplot(kmos1,

pval=TRUE,

pval.coord = c(0, 0.4),

xlab="Survival Months",

ylab="Overall Survival",

legend= "left",

break.x.by=12,

xlim=c(0,96),

pval.size =5,

risk.table=TRUE,

risk.table.height = 0.25,

palette="lancet",

title="OS (n=5,120)",

legend.labs=c("No","Local Surgery","Radical Surgery"),

legend.title="Surgery",

surv.median.line = "hv")

kmcssct<-survfit(Surv(time = aa$Survival,event =aa$CSS==0)~Chemotherapy,data=aa)

summary(kmcssct)

summary(kmcssct,time=c(36,60))

ggsurvplot(kmcssct,

pval=TRUE,

pval.coord = c(0, 0.4),

xlab="Survival Months",

ylab="cancer-Specific Survival",

legend= "left",

break.x.by=12,

xlim=c(0,96),

pval.size =5,

risk.table=TRUE,

risk.table.height = 0.25,

palette="lancet",

title="CSS (n=5,120)",

legend.labs=c("No/Unknown","Yes"),

legend.title="Chemotherapy",

surv.median.line = "hv")

kmos2<-survfit(Surv(time = aa$Survival,event =aa$OS==0)~Chemotherapy,data=aa)

summary(kmos2)

summary(kmos2,time=c(36,60))

ggsurvplot(kmos2,

pval=TRUE,

pval.coord = c(0, 0.4),

xlab="Survival Months",

ylab="Overall Survival",

legend= "left",

break.x.by=12,

xlim=c(0,96),

pval.size =5,

risk.table=TRUE,

risk.table.height = 0.25,

palette="lancet",

title="OS (n=5,120)",

legend.labs=c("No/Unknown","Yes"),

legend.title="Chemotherapy",

surv.median.line = "hv")

########PSM screens matching participants, taking Chemothrapy stratification as example####################

vars <- c("Age","Race","Surgery","Marital", "Radiation","Chemotherapy","brainMeta","liverMeta","lungMeta","Grade", "Gleason","PSA", "TStage","N")

catvars <- c("Age","Race","Surgery","Marital", "Radiation","Chemotherapy","brainMeta","liverMeta",

"lungMeta","Grade", "Gleason","PSA", "TStage","N")

tabletwo <- CreateTableOne(vars = vars,factorVars = catvars,strata="Chemotherapy",data = aa)

table2 <- print(tabletwo, contdigits=3,pDigits =4,showAllLevels = TRUE,

quote = FALSE, noSpace = TRUE, printToggle = FALSE)

tableall2<- CreateTableOne(vars = vars,factorVars = catvars,data = aa)

table00 <- print(tableall2, contdigits=3,pDigits =4,showAllLevels = TRUE, quote = FALSE,

noSpace = TRUE, printToggle = FALSE)

aa$Chemotherapy <- as.factor(aa$Chemotherapy)

m.out <- matchit(Chemotherapy ~ Age + Surgery + Marital+liverMeta+lungMeta +Gleason+N+Grade,

method = "nearest", ratio =1, data = aa,

distance = "logit", replace = FALSE)

summary(m.out)

aamatched<- match.data(m.out)

library(tableone)

vars <- c("Age","Race","Surgery","Marital", "Radiation","Chemotherapy","brainMeta","liverMeta",

"lungMeta","Grade", "Gleason","PSA", "TStage","N")

catvars <-

c("Age","Race","Surgery","Marital", "Radiation","Chemotherapy","brainMeta","liverMeta",

"lungMeta","Grade", "Gleason","PSA", "TStage","N")

table3 <- CreateTableOne(vars = vars,factorVars = catvars,strata="Chemotherapy",

addOverall = TRUE, data = aamatched)

tablematched <- print(table3, showAllLevels = TRUE, quote = FALSE, noSpace = TRUE, printToggle = FALSE)

mm=aamatched

ym<- Surv(time = mm$Survival,event = mm$CSS==0)

Uni_coxm<- function(x){

FMLm <- as.formula(paste0 ("ym~",x))

coxm<- coxph(FMLm,data=mm)

cox1m<-summary(coxm)

HR <- round(cox1m$coefficients[,2],2)

PValue <- round(cox1m$coefficients[,5],3)

CI5 <-round(cox1m$conf.int[,3],2)

CI95 <-round(cox1m$conf.int[,4],2)

Uni_coxm<- data.frame(

names <-rownames(cox1m$conf.int),

'HR' = HR,

'CI5' = CI5,

'CI95' = CI95,

'P' = PValue)

return(Uni_coxm)

}

variable.names<- colnames(mm)[c(1,3:4,12:18,20:21,24:26)];variable.names

library(plyr)

Uni_coxpsm <- lapply(variable.names, Uni_coxm)

Uni_coxpsm<- ldply(Uni_coxpsm, data.frame)

Uni_coxpsm$HR.CI95<-paste0(Uni_coxpsm$HR," (",Uni_coxpsm$CI5,'-',Uni_coxpsm$CI95,")");Uni_coxpsm

library(survival)

ymos<- Surv(time = mm$Survival,event = mm$OS==0)

Uni_coxmos<- function(x){

FMLmos <- as.formula(paste0 ("ymos~",x))

coxmos<- coxph(FMLmos,data=mm)

cox2<-summary(coxmos)

HR <- round(cox2$coefficients[,2],2)

PValue <- round(cox2$coefficients[,5],3)

CI5 <-round(cox2$conf.int[,3],2)

CI95 <-round(cox2$conf.int[,4],2)

Uni_coxmos<- data.frame(

names <-rownames(cox2$conf.int),

'HR' = HR,

'CI5' = CI5,

'CI95' = CI95,

'P' = PValue)

return(Uni_coxmos)

}

Uni_coxpsmos <- lapply(variable.names, Uni_coxmos)

Uni_coxpsmos<- ldply(Uni_coxpsmos, data.frame)

Uni_coxpsmos$HR.CI95<-paste0(Uni_coxpsmos$HR," (",Uni_coxpsmos$CI5,'-',Uni_coxpsmos$CI95,")");Uni_coxpsmos

###Multivariate COX regression after PSM(chemotherapy stratification)#####

mul_coxm<-coxph(Surv(time = mm$Survival,event = mm$CSS==0)~ Age+Race+Marital+Surgery+Radiation+Chemotherapy+Grade+Gleason+PSA+TStage+N+liverMeta+lungMeta+brainMeta,

data=mm)

summary(mul_coxm)

coxcssm<-summary(mul_coxm)

coxcssm$coefficients

coxcssm$conf.int

mul_HR<- round(coxcssm$coefficients[,2],2)

mul_PValue<- round(coxcssm$coefficients[,5],4)

mul_CI1<-round(coxcssm$conf.int[,3],2)

mul_CI2<-round(coxcssm$conf.int[,4],2)

mul_CI95<-paste(mul_CI1,'-',mul_CI2)

mul_coxcssm <- data.frame("HR" =mul_HR,

"CI95" =mul_CI95,

"P"=mul_PValue);mul_coxm

mul_coxmos<-coxph(Surv(time = mm$Survival,event = mm$OS==0)~ Age+Race+Marital+Surgery+Radiation+Chemotherapy+Grade+Gleason+PSA+TStage+N+liverMeta+lungMeta+brainMeta,data=mm)

summary(mul_coxmos)

coxcssm2<-summary(mul_coxmos)

coxcssm2$coefficients

coxcssm2$conf.int

mul_HR<- round(coxcssm2$coefficients[,2],2)

mul_PValue<- round(coxcssm2$coefficients[,5],4)

mul_CI1<-round(coxcssm2$conf.int[,3],2)

mul_CI2<-round(coxcssm2$conf.int[,4],2)

mul_CI95<-paste(mul_CI1,'-',mul_CI2)

mul_coxcssm2 <- data.frame("HR" =mul_HR,

"CI95" =mul_CI95,

"P"=mul_PValue);mul_coxmos

###Nomogram、Calibration Curve######

nomo<-datadist(aa)

options(datadist='nomo')

nomo0 <- cph(Surv(time = aa$Survival,event =aa$CSS==0)~Age+Race+Marital+Surgery+Chemotherapy+Grade+Gleason+PSA+

TStage+liverMeta+lungMeta,

x=T,y=T,

data=aa,

surv=T,

time.inc = 12*3);nomo0

Cindex0 <- rcorrcens(Surv(as.numeric(aa$Survival),aa$CSS==0)~predict(nomo0))

surv <- Survival(nomo0)

surv1 <- function(x)surv(12*3,lp=x)

surv2 <- function(x)surv(12*5,lp=x)

nomo1<-nomogram(nomo0,

fun=list(surv1,surv2),

funlabel=c('3-year Cancer-Spefic Survival',

'5-year Cancer-Specific Survival'),

lp =F,

maxscale=100,

fun.at=c("0.99","0.95",'0.9','0.8',

'0.7','0.6','0.5','0.4',

'0.3','0.2','0.1')

);plot(nomo1,col.grid = gray(c(0.8,0.95)), xfrac = 0.35,cex.var = 1,cex.axis=0.8, tcl=-0.3,lmgp=0.3)

nomoos <- cph(Surv(time = aa$Survival,event =aa$OS==0)~Age+Race+Marital+Grade+Gleason+PSA+Surgery+

Chemotherapy+TStage+liverMeta+lungMeta+brainMeta,

x=T,y=T,

data=aa,

surv=T,

time.inc = 12*3);nomoos

Cindexos <- rcorrcens(Surv(as.numeric(aa$Survival),aa$OS==0)~predict(nomoos))

survos <- Survival(nomoos)

survos1 <- function(x)survos(12*3,lp=x)

survos2 <- function(x)survos(12*5,lp=x)

nomo2<-nomogram(nomoos,

fun=list(survos1,survos2),

funlabel=c('3-year Overall Survival',

'5-year Overall Survival'),

lp =F,

maxscale=100,

fun.at=c("0.99","0.95",'0.9','0.8',

'0.7','0.6','0.5','0.4',

'0.3','0.2','0.1')

);plot(nomo2,col.grid = gray(c(0.8,0.95)), xfrac = 0.35,cex.var = 1,cex.axis=0.8, tcl=-0.3,lmgp=0.3)

p <- calibrate(nomo0,

cmethod='KM',

method='boot',

u=12*3,

m=1700,

B=1000)

plot(p,

add=F,

conf.int=T,

subtitles = F,

cex.subtitles=0.8,

lwd=2,

lty=1,

errbar.col="blue",

xlim=c(0.1,0.8),

ylim=c(0.1,0.8),

xlab="Nomogram Predicted 3-year Cancer-Specific Survival in Training Cohort",

ylab="Actual 3-year Survival",

col="blue")

p <- calibrate(nomoos,

cmethod='KM',

method='boot',

u=12*3,

m=1700,

B=1000)

plot(p,

add=F,

conf.int=T,

subtitles = F,

cex.subtitles=0.8,

lwd=2,

lty=1,

errbar.col="blue",

xlim=c(0.1,0.8),

ylim=c(0.1,0.8),

xlab="Nomogram Predicted 3-year Overall Survival in Training Cohort",

ylab="Actual 3-year Survival",

col="blue")

#####Decision Curve##############

f1 <- coxph(Surv(Survival, CSS==0) ~Age+Race+Marital+Gleason+PSA+Surgery+Grade+Chemotherapy+TStage+liverMeta+lungMeta,

data = aa)

aa$Nomogram= c(1- (summary(survfit(f1, aa), times=36)$surv))

dca(Surv(Survival, CSS==0)~Nomogram,

data = aa,

time = 36,

thresholds = 1:100/ 100) %>%

standardized_net_benefit() %>%

plot(smooth = T)

f2 <- coxph(Surv(Survival, CSS==0) ~Age+Race+Marital+Gleason+PSA+Surgery+Grade+Chemotherapy+TStage+liverMeta+lungMeta,

data = aa)

aa$Nomogram= c(1- (summary(survfit(f2, aa), times=60)$surv))

dca(Surv(Survival, CSS==0)~Nomogram,

data = aa,

time = 60,

thresholds = 1:100/ 100) %>%

standardized_net_benefit() %>%

plot(smooth = T)

f3 <- coxph(Surv(Survival, OS==0) ~Age+Race+Marital+Gleason+PSA+Surgery+Grade+Chemotherapy+

TStage+liverMeta+lungMeta+brainMeta,

data = aa)

aa$Nomogram= c(1- (summary(survfit(f3, aa), times=36)$surv))

dca(Surv(Survival, OS==0)~Nomogram,

data = aa,

time = 36,

thresholds = 1:100/ 100) %>%

standardized_net_benefit() %>%

plot(smooth = T)

f4 <- coxph(Surv(Survival, OS==0) ~Age+Race+Marital+Gleason+PSA+Surgery+Grade+Chemotherapy+

TStage+liverMeta+lungMeta+brainMeta,

data = aa)

aa$Nomogram= c(1- (summary(survfit(f4, aa), times=60)$surv))

dca(Surv(Survival, OS==0)~Nomogram,

data = aa,

time = 60,

thresholds = 1:100/ 100) %>%

standardized_net_benefit() %>%

plot(smooth = T)
